# Research Progress on Pathogenesis and Prevention of Avian Leukosis Virus J Subgroup (ALV-J)

**DOI:** 10.3390/vetsci13020152

**Published:** 2026-02-04

**Authors:** Xinyu Liu, Xi Lan

**Affiliations:** Chongqing Key Laboratory of Herbivore Science, College of Animal Science and Technology, Southwest University, Chongqing 400700, China; liuxinyu06162006@126.com

**Keywords:** avian leukemia virus J subgroup, pathogenic characteristics, cell infection and proliferation, host resistance, disease-resistant breeding

## Abstract

As a major retrovirus threatening global poultry farming, Avian Leukosis Virus Subgroup J (ALV-J) has expanded its host range since discovery, extending from conventional broilers to layer chickens and native breeds. Its diverse oncogenic manifestations, including myeloid leukemia, hemangiomas, and tumors of immune and visceral organs, have led to increased mortality, reduced productivity, and substantial economic losses in the poultry industry. Based on the current body of literature, this review summarizes and synthesizes advances in the etiological characteristics, infection and pathogenic mechanisms, host resistance, and research progress in prevention and control of ALV-J. Accumulating evidence indicates that viral evolution driven by mutations and recombination—particularly in the *env* gene and *LTR* regions—plays a central role in host range expansion, tumor diversity, and immune evasion. Current studies consistently demonstrate that host resistance to ALV-J is a multifactorial process involving genetic polymorphism, innate immune responses, and cellular autonomous defense systems. In this context, recent advances in disease-resistant breeding highlight CRISPR-Cas9-mediated gene editing as a promising strategy for blocking viral entry or replication. Despite these advances, major gaps remain, including an incomplete understanding of virus–host interaction networks, limited insight into co-infection-mediated synergistic pathogenicity, the absence of effective vaccines, and insufficient large-scale epidemiological surveillance and purification systems. Addressing these challenges will be critical for the development of integrated prevention strategies and the sustainable control of ALV-J in poultry production.

## Simple Summary

Avian leukosis virus subgroup J is a contagious virus that causes cancer-like diseases in chickens and has become a serious problem for poultry farming worldwide. Since it was first discovered, this virus has spread from meat-type chickens to egg-laying and local chicken breeds, leading to major economic losses due to reduced productivity and increased mortality. This review aims to clearly explain how the virus infects chickens, how it causes disease, and why it is difficult to control. We summarize recent research showing that some chickens naturally carry genes that make them more resistant to infection, and scientists are using this knowledge to develop disease-resistant chickens through selective breeding and modern gene-editing methods. The review also discusses how the virus can weaken the immune system, evolve rapidly, and sometimes escape control measures, which makes prevention challenging. In addition, we highlight progress and remaining gaps in developing faster diagnostic tools, safer vaccines, and effective farm-level control strategies. Finally, we emphasize the importance of long-term monitoring systems and clean breeding programs to stop the virus from spreading between generations. Overall, this work helps farmers, researchers, and policymakers better understand this disease and supports the development of healthier poultry populations, safer food production, and more sustainable agricultural practices that benefit society as a whole.

## 1. Introduction

Avian leukosis is an infectious disease induced by Avian Leukosis Virus (ALV). The term describes a group of diseases caused by ALV, primarily malignant tumours in the hematopoietic tissues of affected poultry [1]. Since its emergence, ALV infection has been reported in major poultry-producing regions worldwide, particularly in countries with intensive breeding and large-scale production systems [1,2,3]. ALV-associated diseases result in increased mortality, reduced growth performance, decreased egg production, and the culling of infected flocks, thereby causing substantial economic losses to the global poultry industry [1,2].

Currently, research on ALV has focused mainly on the J subgroup (ALV-J) because of their pathogenicity and the broad range of host species they can infect [1,2]. The first recorded case of ALV-J was in 1989, when the virus was isolated from a flock of broiler chickens in the United Kingdom, and the virus has now spread around the world [4]. ALV-J is highly pathogenic in broilers, primarily causing malignant tumors known as myelocytomas (neoplasms of myeloid cells) [4,5]. Moreover, the virus can cross species barriers and have been shown to produce multiple pathologies such as angiomas and hepatosplenomegaly in layers and local breed chickens [3,6,7]. Compared with other ALV subgroups, ALV-J exhibits greater genetic variability and more diverse oncogenic patterns, which contribute to its enhanced adaptability and re-emergence even in purified flocks. These characteristics pose serious challenges to disease control and could severely affect the sustainable development of the poultry industry [2,6,8,9].

ALV-J infection results from complex interactions between the virus and host cells, involving multiple stages such as viral entry, integration of the viral genome into the host genome, and replication and release of progeny viruses. At the entry stage, chicken Na^+^/H^+^ exchanger 1 (chNHE1) has been identified as the primary cellular receptor for ALV-J, and specific mutations in the viral envelope protein gp85 are closely associated with receptor binding affinity and host range expansion. Following entry, ALV-J infection activates multiple host signaling pathways, including Wnt/β-catenin, NF-κB, and ERK/AP-1 pathways, which contribute to viral replication, cell proliferation, and tumorigenesis. In addition, accumulating evidence indicates that ALV-J employs diverse immune evasion strategies, such as modulation of innate immune signaling, microRNA-mediated gene regulation, and epigenetic reprogramming, to facilitate persistent infection and tumor development [1,2]. During each of these phases, there are complex and detailed molecular interactions. Because of the advancements in molecular biology, research has increased its understanding of the pathogenesis and the intracellular proliferation of ALV-J as well as the host’s defense mechanisms [10,11,12]. This knowledge is essential for developing breeding and disease management strategies to produce resistant birds [13,14]. This paper summarises the most recent research evaluating the core pathogenesis of ALV-J to provide a detailed foundation for further research on effective control and prevention of this virus.

## 2. Pathogenic Characteristics of ALV-J

The genus from which ALV-J was derived (Alpharetrovirus family) contains typical retroviruses, but ALV-J has distinct genetic characteristics, biological classifications, and pathogenesis/epidemiology from those in that genus. As ALV-J adapts to its current or new hosts, it continues to propagate its evolutionary traits, creating the greatest opportunity for modifications to occur that enable transmissibility and pathogenesis.

### 2.1. Classification and Properties of ALV-J

Although ALV-J has been traditionally classified as an exogenous virus since it can be transmitted both horizontal and vertically inside poultry, it is categorized quite differently than the earlier classification of the five subgroups A to E, in that it does not belong to the endogenous form of ALV found in many species. The physical distribution of ALV-J among poultry is primarily through horizontal transmission by means of contaminated environment, water or feed, whereas vertical transmission occurs when a hen passes the virus onto her offspring via infectious eggs, causing congenital infection in chicks [4]. This is one of the two major reasons that allow ALV-J to remain endemic in poultry flocks and prevent efforts to eradicate the disease. Unlike the endogenous forms of ALV, the exogenous form of ALV-J does not integrate into the germline of the host’s genome, but after it infects its host, it will integrate into the somatic cell genome for many years, permitting long-term latency and subsequently replication [15].

### 2.2. Structure and Genetic Characteristics of ALV-J

Genotypically, ALV-J possesses a positive-sense single-stranded RNA genome of approximately 7600 nt, which is structured as a 5′-LTR-gag-pol-env-3′-LTR. The various functional and variation properties of the genes and regulatory regions in this genome greatly influence the pathogenicity of the virus, the host range of the virus and the replication ability of the virus [8].

#### 2.2.1. *Env* Gene

The *env* gene is a key component to classify ALV-J into different subgroups and is responsible for the production of the envelope proteins, gp85 and gp37. The receptor for gp85 was determined to be host cell surface receptor; however, the amino acids of gp85 only shared 40% homology with the amino acids of subgroups A-E [1], which indicates that gp85 has different antigenicity from subgroups A-E and, therefore, limited or no effective cross-protective immunity has been demonstrated [16]. Highly variable *env* genes of ALV-J strains also exist from the various species of hosts. An example of this variability can be observed in the *env* gene sequences of ALV-J isolates from Wuhua and Huang chickens in China, which show high nucleotide sequence identity and are closely related to representative domestic strains from other countries. In contrast, ALV-J strains isolated from Malaysia and Europe/America display greater sequence divergence in the *env* gene, with reported nucleotide identity ranging from approximately 88.6% to 99.4%, based on comparative analyses of field isolates [6]. In addition to the considerable homology differences of the *env* gene among host species, highly variable regions are present within the gp85 surface subunit (SU), specifically at amino acid positions 38–131 and 159–283. These regions constitute a bipartite receptor-binding domain unique to ALV-J and mediate interaction with the host cell surface receptor, chicken sodium/hydrogen exchanger 1 (chNHE1) [15]. Certain strains of ALV-J that originated from laying hens show deleted or inserted amino acids within some of the highly variable regions located in the *gp85* gene. One example of these highly variable strains is strain JS09GY3, which contains an 11-bp insertion and this may result in altered antigenicity and receptor binding ability of the virus and, therefore, is also a factor in changed pathogenicity [7].

**Figure 1:**
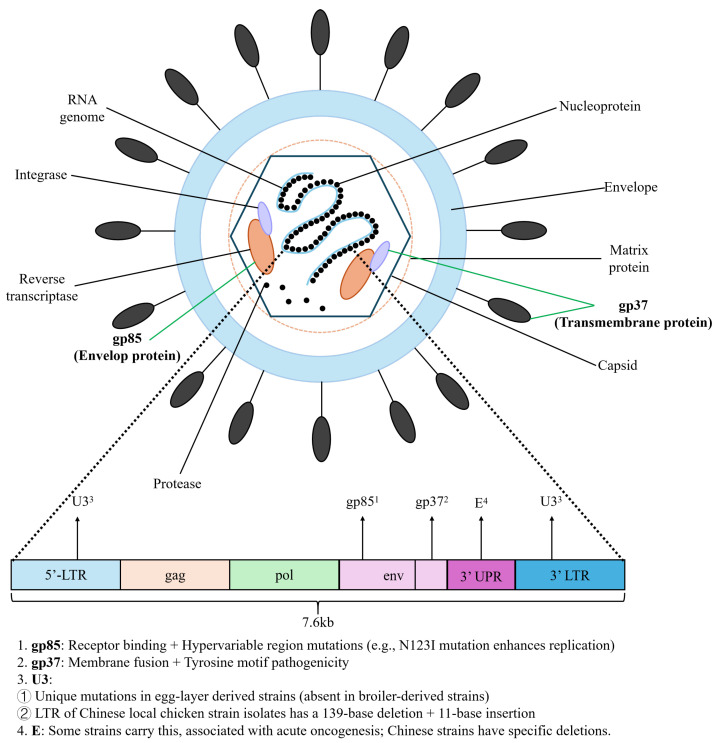
Structure and Genetic Characteristics of the ALV-J.

The gp37 transmembrane (TM) subunit mediates membrane fusion between the viral envelope and the host cell membrane during ALV-J entry. The C-terminal tyrosine motif of gp37 plays a direct role in determining the pathogenicity of the virus. Based on differences in the tyrosine motifs of gp37 of ALV-J strains, we can classify the strains into three types: The inhibitory type (possess ITIM motif only), the bifunctional type (possess both ITIM and YxxM motifs), and the active type (possess YxxM and ITAM-like motifs) [6,8,17]. Active type strains have greater replicative ability and pathogenicity due to their ability to effectively inhibit the host immune response, whereas the inhibitive type strains are readily cleared from the host by the host’s immune system [17] (Figure 1).

#### 2.2.2. *Gag*/*Pol* Genes

The *gag* and *pol* genes, unlike the *env* gene, are much more conserved than the *env* gene, with *gag* and *pol* genes from ALV subgroups A-E sharing 96–97% homology with other groups. The gag gene produces p27, which is the core viral protein and is a good diagnostic tool for identifying ALV-J infected birds. The core virus proteins can be detected by antigen capture ELISA [18,19]. The *pol* gene codes for enzymes, including reverse transcriptase and integrase enzymes, which are responsible for the processes of reverse transcription of viral RNA and integration of genomic cDNA into host genomic DNA. Because of the level of conservation of *pol* genes, the *pol* gene allows ALV-J to replicate effectively in a variety of different host cells. For this reason, the *pol* gene is one of the more important functional genes in the viral life cycle [8] (Figure 1).

#### 2.2.3. *LTR* Region

The Long Terminal Repeat regions located at the 5′ and 3′ ends of the genome form the *LTR* regions. There are two *LTR* regions, one located at each end of the genome. Both of these regions are similar but different in function, with the 3′*LTR* serving as the promoter region and the 5′*LTR* serving as the transcription terminator region. The viral LTRs contain numerous binding sites for transcription factors that regulate the expression of the viral genes. The mechanisms of regulation of virus LTRs are poorly understood at present [4]. The LTR promoter elements have similarities to cellular promoter sequences, indicating that ALV-J transcribes its genes using cellular machinery [15] (Figure 1).

#### 2.2.4. E Component

In the past, The E component of ALV-J has been discovered only in Rous Sarcoma Virus (RSV), a virus known for its ability to cause cancer in chickens. However, recent studies have shown that some prevalent strains of ALV-J isolated from commercial laying hens (e.g., Hy-Line Brown or similar breeds commonly used in intensive farming systems) and indigenous chicken breeds in China (e.g., Wuhua chicken from Anhui Province) also contain complete E components. For instance, the strain SCDY1, isolated from a grandparent layer flock, harbors a full-length E element [8]. Therefore, the presence of complete E components on these strains may help explain the highly Oncogenic (cancer causing) properties that they possess (recently isolated from chickens) [7]. The E components of Chinese strains of ALV-J have undergone deletions (networks) to adapt to local populations. It has been shown that the E components of the 4817 (U.S.) have approximately 99% homology with the majority of E components found in Chinese strains and therefore these deleted sections may represent an important point in the evolution of ALV-J as it adapted to its environment for both pathogenicity and transmissibility (to new hosts) in China [4] (Figure 1).

### 2.3. Pathogenicity of ALV-J

ALV-J’s pathogenic character is defined by three major components: significant host specificity; a wide range of tumor types; and immunosuppression. The range of susceptible hosts has increased from broiler chickens to include both laying hens and many local breeds of chickens, therefore increasing the diversity of pathological effects produced, including but not limited to myelocytomas, angiomas and immune organ tumors. In addition, ALV-J is a rapidly evolving virus and continues to mutate as it replicates, which greatly increases its ability to replicate and be transmitted within a single generation. Concurrently, ALV-J exhibits pronounced tropism for immune organs, leading to extensive suppression of the host immune response. Immune suppression occurs through interference with the efficacy of vaccines, apoptosis and epigenetic changes, which in turn increase the likelihood of disease and provide a continuous threat to the poultry industry [1].

#### 2.3.1. Host Specificity and Expansion of Host Range

**Figure 2:**
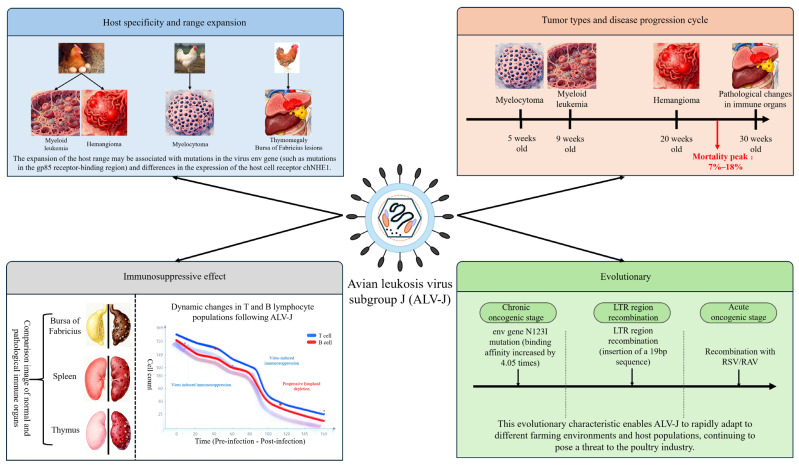
Pathogenicity of ALV-J.

Initial studies suggested that ALV-J was limited to broilers (including both breeding and commercial chickens), however; this incorrect assumption has led to evidence suggesting that the host range of ALV-J continues to expand as it is shown to have adapted to multiple different breeds of chickens (for example, layers, and locally raised birds such as Wuhua, yellow, and Tibetan) [6]; therefore, ALV-J infection leads to various disease processes and degrees of pathogenicity depending on the affected host. For example, broilers are mainly affected with myeloma when infected with ALV-J, while layers may develop mainly myeloid leukaemia and hemangioma upon infection. Additionally, yellow chickens and other local breeds may develop both the classic neoplasms of the liver, spleen, and kidneys as well as enlargement of their thymus and proliferation of the bursa of Fabricius due to infection with ALV-J [3]. It is hypothesized that host range expansion may be attributed to mutations in the viral *env* gene (for example, mutations in the gp85 receptor-binding domain) and variable chNHE1 host receptor expression. Through genetic mutation, the virus can adapt to the cellular environment of a particular host and therefore has the capacity to transmit between species [20] (Figure 2).

#### 2.3.2. Tumor Types and Disease Cycle

The avian leukosis virus subtype J (ALV-J) has been associated with many types of tumors, with the myeloid tumors being the most recognized. The disease generally appears around 5 weeks of age in chickens; however, most chickens will start showing signs 9 weeks after being infected. The highest rates of mortality due to this disease are usually seen by 20 weeks [4]. ALV-J’s types of induced tumors are diversifying as it continues to adapt to its host. Besides myeloid leukemia, in hens that produce eggs, hemangiomas have developed (around 10% of all cases). Also, tumors have occurred on the skin and viscera of chickens (i.e., liver, spleen), and appear red or dark red protrusions that are susceptible to rupture and bleeding, thus leading to the death of chickens [21]. In indigenous breeds, such as yellow chickens, immune organs, such as the thymus and bursa of Fabricius, can have tumors. Co-infection with other viruses, such as Reticuloendotheliosis Virus (REV) can further complicate the variety of tumors resulting from ALV-J and make the diagnosis and control of the disease more challenging [3]. The ALV-J disease cycle is relatively long, with weeks to several months likely between infection and onset of clinical signs. The specific length of the ALV-J disease cycle varies between strains, as evidenced by yellow chickens developing disease signs by 16 weeks; with the peak mortality rates occurring between 20 and 30 weeks (ranging from 7% to 18%), which is dramatically higher than that seen with the classical disease cycle in broiler chickens [3] (Figure 2).

#### 2.3.3. Immunosuppressive Effect

ALV-J infection has a severe negative impact on the immune system of the host, decreasing their resistance to other infections and predisposing them to the development of secondary infections [10]. The immune suppression induced by the virus occurs through many different mechanisms including inhibition of antibody production in response to vaccines (e.g., Newcastle Disease and H5-type Avian Influenza), resulting in failure of the vaccine to provide the intended protection from the disease. The virus also infects tissues associated with the immune system (thymus, bursa of Fabricius, spleen) disrupting the growth and function of immune cells that include thymic lymphocytes and bursal follicular cells, thereby decreasing the overall numbers of T cells and B cells [3]. The virus can modulate patterns of immune-related signaling pathways within the host (e.g., NF-κB and JAK-STAT pathways), which will result in the down-regulation of the production of cytokines (including IFN), thus negatively impacting the host’s innate immune response [22]. The immune suppression caused by ALV-J virus also may be further increased by down-regulatory epigenetic mechanisms (e.g., methylation at the M6 position on Adenosine and specific miRNA-related) in order to set conditions for both enhanced viral growth and spread [23] (Figure 2).

#### 2.3.4. Evolutionary Characteristics

Long-Term Transmission of ALV-J has seen the virus evolve from Chronic Tumorigenesis to Acute Tumorigenesis, with increased Infectivity and pathogenicity markings over time. This evolutionary trajectory is characterized by genetic optimization, recombination events, and mutations that enhance viral fitness, as detailed in the following sections [4]. In particular, ALV-J has developed its genome through Continuous Genetic Optimization by Genetic Mutations (e.g., Variants of *env* Gene; *LTR* Region), as well as by way of Genetic Recombination (e.g., Recombination with RSV/RAV-1/2 Viruses) [7]. This process has led to increased efficiency of Replication and Pathogenicity of ALV-J. Additionally, as described in previous reports, ALV-J Strains from laying hens through Recombination with RSV-SRB/RAV-1/2 have incorporated an additional 19-bp foreign Sequence into the primer binding site (PBS). This Inclusion significantly enhanced the ALV-J Replication Capacity. For example, N123I Mutation (conversion of N’sgp85 RBD) has increased binding capacity of the ALV-J Virus to the chNHE1 Receptor 4.05 times, with a 10× in vitro increase in Viral Titer and a 13.4× increase in Viral Load in vivo, which consequently, has led to both faster Viral Spread and greater severity of Damage to the Host [20]. ALV-J’s predicting trait allows for rapid adaptation to new farming environments and host populations, thus continuing to pose a significant Threat to the Poultry Industry [17] (Figure 2).

### 2.4. Epidemiological Characteristics of ALV-J

The epidemiology of ALV-J is characterized by its persistence, adaptability, and heterogeneous impact across different hosts and regions, which collectively complicate its control.

#### 2.4.1. Covert Nature

Some ALV-J–infected chickens remain clinically asymptomatic yet mount an antibody response, despite the absence of detectable virus in blood or fecal samples, and may still transmit the virus [6]. This latent infection is likely attributable to the virus’s ability to evade host immune responses through mechanisms such as miRNA modulation, epigenetic regulation, and alterations in host immune status. The ALV-J virus can establish a persistent infection in host cells. Following initial infection and integration into the host genome, the provirus may enter a state of low-level replication or transcriptional silence, evading immune clearance. This state can be maintained for extended periods. However, under conditions of host immunosuppression or other physiological stresses, the transcriptional activity of the integrated provirus may be upregulated, leading to enhanced viral replication and shedding, and potentially triggering clinical disease or facilitating transmission. In addition to latent infection, ALV-J can be vertically transmitted through the embryo, resulting in congenitally infected chicks that may remain asymptomatic for weeks or months, thereby complicating outbreak detection and control [3,23].

#### 2.4.2. Regional Variability

ALV-J strains exhibit significant genetic diversity that often correlates with geographic origin, reflecting localized evolution and adaptation. This regionalization is driven by factors such as local farming practices, movement of poultry stock, and genetic recombination with other viruses (e.g., RSV). Consequently, dominant strains and their associated disease profiles (e.g., myelocytoma vs. hemangioma predominance) can vary between regions, necessitating region-specific surveillance and diagnostic approaches [6,17].

#### 2.4.3. Breed Differences

Susceptibility to ALV-J varies among chicken breeds, with white-feathered broilers being the most susceptible and exhibiting higher morbidity and mortality than layers or native breeds. Native breeds, such as Tibetan chickens, have adapted to their environments over time and developed resistance mechanisms, including the overexpression of genes such as *RFX1* and *VCAM1*, which inhibit ALV-J replication and tumor formation, resulting in reduced viral load and pathology [24]. Identifying breeds and key resistance genes associated with ALV-J resistance would provide valuable genetic resources for poultry breeding programs, enabling the development of chickens with enhanced innate resistance and contributing to reduced viral transmission [11].

## 3. ALV-J Infection of Cells and Intracellular Proliferation Process

The ALV-J virus Invades its host’s cells in a stage dependent manner that involves several steps, including how the virus identifies its host cell; how it Invades; how it Copies its genome as Reverse Transcription; how the virus integrates into its host cell; how the virus assembles and ultimately releases copies of itself back into the environment, and More. All of these steps will vary from one strain of ALV-J to another strain, as well as between species of Host (such as chicken vs. human). Each of these “Steps” will be Infected by both viral and cellular factors involved in Signalling Pathways.

### 3.1. Susceptible Cell Types for ALV-J

Infection with ALV-J is specific to certain cell types, which can only be infected if the host cell expresses a specific receptor for ALV-J, chNHE1. However, the types of cells susceptible to ALV-J infection and that are able to support high levels of virus proliferation vary widely based on additional factors such as the amount of chNHE1 on their surface membranes, the metabolic and immune characteristics of those cell types and the expression of chNHE1 [15].

#### 3.1.1. In Vitro Cultured Cells

**Figure 3:**
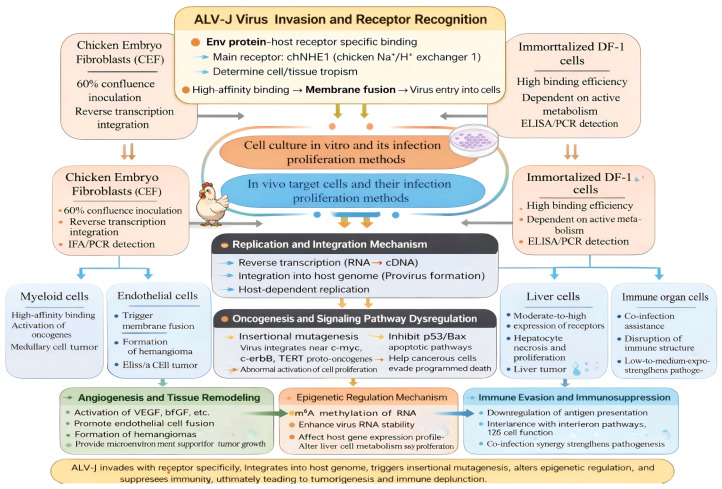
Susceptible cell types and infection and key mechanisms of ALV-J.

Chicken embryo fibroblast (CEF) cells from the specific pathogen-free (SPF) chick embryo have served as a standard cell type to study, isolate, and propagate avian leukosis virus subgroup J (ALV-J) in vitro [4]. In order for the CEF to infect the host cell and replicate effectively, the CEF must be in a state of confluence of approximately 60% when placed onto tissue culture dishes that have been coated with 0.25% trypsin [4]. The presence of the ALV-J in the CEF can be determined by indirect immunofluorescent assays (IFA) that detect the expression of the viral gp85 protein, as well as by amplifying proviral DNA using polymerase chain reaction (PCR) [4]. Immortalized DF-1 chicken embryo fibroblast (CEF) cells have also proven to be an appropriate cell type for ALV-J research, as these cells are free from the presence of endogenous ALV and maintain a stable growth pattern [7]. Therefore, DF-1 cells can be used to carry out culturing of the virus over extended periods of time for the purposes of titer determinations and drug testing [7]. When ALV-J infects DF-1 cells, a full viral cycle of infection and proliferation takes place in approximately 7–9 days from the time of infection. After multiple freeze–thaw cycles, the DF-1 cells can be used for antigen capture enzyme linked immunosorbent assay (ELISA) and PCR to confirm the presence of viral proteins and proviral DNA, and the high-efficiency replication of ALV-J in DF-1 cells [7] (Figure 3).

#### 3.1.2. In Vivo Target Cells

In vitro research has shown that ALV-J (Avian leukosis virus—serotype J) can be isolated from multiple types of target cell types found in a wide variety of bird breeds [21]. This polyclonal nature of infection is due to the following factors: host breed, type of viral strain infecting the recipient, the location of the infection (i.e., bone marrow vs. other locations), and the host immune system’s response to the invader virus. While infected target cells will induce different pathologies and tumor types when infected with ALV-J depending on the factors mentioned, it has been shown that the principal target types are; myeloid cells, endothelial cells, immune organ cells (i.e., thymus, bursa of Fabricius, spleen), and liver cells.

1. Myeloid Cells

Myeloid cells in vivo are the primary target cell type infected by ALV-J. The virus infects the immature (pre) myeloid teardrop-shaped precursor type cells found in the bone marrow, then induces them to undergo uncontrolled cell division (abnormal proliferation and differentiation) that eventually result in myelocytomas, which are tumors that are a characteristic pathological finding in broiler chickens infected with ALV-J [4]. Myeloid cells express very high levels of chNHE1 (chicken sodium/hydrogen exchangers) receptors, which allow easy access to, and invade, the cells by the virus. Following entry into myeloid cells, the viral RNA genome undergoes reverse transcription in the cytoplasm to generate proviral DNA, which is subsequently transported into the nucleus and integrated into the host genome. This integration can activate proto-oncogenes (e.g., c-myc) and disrupt tumor suppressor pathways (e.g., p53), thereby promoting malignant transformation of myeloid cells [25] (Figure 3).

2. Endothelial Cells

Endothelial cells are the primary target cell type for ALV-J in laying hens. The virus induces uncontrolled cell division and abnormal proliferation of these cells, leading to the formation of angiomas [21]. Endothelial cells express high levels of the chNHE1 receptor on the surface of the cell, and it is postulated that the virus induces new blood vessel formation (angiogenesis) via modulating the modifications made to the signaling pathways (i.e., via vascular endothelial growth factor (VEGF)). Hence, it is believed that this is the molecular mechanism responsible for the formation of angiomas in the laying hens infected with ALV-J [26] (Figure 3).

3. Immune Organ Cells

ALV-J can infect immune organ cell types, including the thymus, bursa of Fabricius, and spleen; all of which are composed of a variety of immune organ-specific cells (e.g., thymic lymphocytes, bursal follicular cells, splenic macrophages) [10]. Infection of these cells disrupts the structure of the immune organ, as well as the immune organ cells themselves, resulting in inhibition of the immune system from performing its normal activities [3]. The immune cells in the immune organs are highly susceptible to the immunosuppressive effects of the virus due to exaggerated normal apoptosis (death) and tumorigenesis, which leads to inhibition of normal immune function (Figure 3).

4. Liver Cells

The liver is one of the principal target organs for infection with the ALV-J virus. The virus promotes necrosis of the hepatocytes, the liver cells, inducing proliferation of the hepatocytes, leading to liver tumors [27]. The liver cells have a very high rate of metabolism, thus providing an abundance of nutrients needed by the virus to replicate. High levels of chNHE1 receptors are found on the surface of the hepatocytes, allowing easy access to and invasion of the cells by the virus. Some strains of the ALV-J virus have been found to promote replication of the virus and tumor development by modulating the epigenetic state of the liver cells, via m6A (N6-methyladenosine) methylation [23] (Figure 3).

### 3.2. Infection and Proliferation Process of ALV-J

Infection of host cells with ALV-J occurs in a series of regulated steps. Virus entry starts with the interaction of glycoprotein gp85 on the viral envelope with the host cell receptor, chNHE1, leading to fusion of both membranes and entry of the viral genome into the cytoplasm. During reverse transcription and integration, viral RNA is reverse transcribed to produce proviral DNA via reverse transcriptase, followed by random integration into the host’s DNA. This step is regulated by both host cell factors (signal transduction) and epigenetic modifications to host DNA (e.g., chemical changes to DNA), in addition to being controlled by the host cell cycle. During the assembly and release step, the virus utilizes the transcriptional and translational machinery of its host to produce viral proteins, assemble virions, and eventually release themselves through budding. Budding is facilitated by the ability of the virus to manipulate the host’s cytoskeleton and to induce cell death. Overall, these steps illustrate how thoroughly the virus “takes over” and controls various processes in its host cell [4].

#### 3.2.1. Phase of Invasion

ALV-J invasion is initiated by the interaction between the viral envelope protein gp85 and the host cell surface receptor chNHE1, mediated through a bipartite gp85 binding domain (amino acids 38–131 and 159–283) that recognizes key residues on the extracellular loop 1 of chNHE1 (A30, V33, W38, and E39), forming a stable virus–receptor complex [15]. This binding triggers conformational changes in gp85 that expose the gp37 fusion peptide, enabling fusion of the viral envelope with the host cell membrane and release of viral RNA into the cytosol. The efficiency of viral entry is influenced by multiple factors, including amino acid substitutions in gp85 (e.g., N123I), which enhance receptor affinity [20], as well as the expression level and modification status of chNHE1, with higher expression conferring increased susceptibility. Conversely, cellular differentiation processes may alter chNHE1 structure or expression and inhibit viral entry [10]. In addition, co-infection with other viruses, such as REV, can facilitate early ALV-J attachment by modulating host cell integrin expression, thereby promoting viral entry [28] (Figure 3).

#### 3.2.2. Reverse Transcription and Integration

Following entry, the viral RNA genome is reverse-transcribed into double-stranded cDNA in the cytoplasm by reverse transcriptase encoded by the viral *pol* gene [8]. This process involves synthesis of a negative-strand cDNA from the RNA template, degradation of the RNA, and subsequent synthesis of the positive strand, resulting in proviral DNA. The proviral cDNA is then randomly integrated into the host genome by integrase, potentially disrupting genomic structure and activating proto-oncogenes or repressing tumor suppressor genes, thereby contributing to malignant transformation [25]. Host epigenetic mechanisms and signaling pathways regulate proviral integration and expression [29]; notably, ALV-J activates the Wnt/β-catenin pathway, promoting nuclear translocation of β-catenin and enhancing viral LTR-driven transcription [29]. Additionally, ALV-J modulates host epigenetic machinery, including m6A RNA methylation and DNA methylation, to create a cellular environment favorable for viral replication [23]. Viral integration and replication are further influenced by the host cell cycle, with S-phase cells being more permissive than G1-phase cells; ALV-J facilitates G1-to-S phase progression by activating cell cycle regulators such as Cyclin D1, thereby supporting efficient viral replication [30] (Figure 3).

#### 3.2.3. Viral Assembly and Release of Progeny Virus

Viral RNA is transcribed from proviral DNA in the host cell nucleus using the host transcriptional machinery and serves both as genomic RNA for viral replication and as mRNA for the synthesis of the major viral proteins (gag, pol, and env) [8]. Gag forms the structural core of the virus, Pol encodes enzymes including reverse transcriptase and integrase, and Env proteins (gp85 and gp37) are synthesized separately and transported to the host cell membrane, likely via lipid rafts, where they assemble with the viral core to form mature progeny virions. Fully assembled viruses are released from host cells by budding and subsequently infect neighboring cells [31]. This release process depends on host cytoskeletal organization and associated signaling pathways; for example, ALV-J exploits cytoskeletal remodeling, and co-infection with REV enhances progeny virus release by upregulating NCKAP1 expression, thereby promoting viral dissemination [18]. In addition, the host cell undergoing apoptosis also plays a role in progeny virus release, as the virus may suppress apoptosis through the upregulation of Bcl-2, a key anti-apoptotic protein that inhibits the activation of the caspase cascade, and the downregulation of Caspase-3/9 expression; thus, by delaying host cell apoptosis, the number of progeny virus produced can be extended [30] (Figure 3).

It should be noted that most mechanistic studies on ALV-J infection and tumorigenesis are currently based on in vitro models, particularly DF-1 and chicken embryo fibroblast (CEF) cells. While these systems are valuable for dissecting virus–host interactions at the molecular level, they lack the complexity of the in vivo immune microenvironment, do not fully reflect tissue-specific viral tropism, and fail to recapitulate the multistep process of tumorigenesis observed in infected chickens. Therefore, conclusions drawn from cell culture–based studies should be interpreted with caution, and further validation using in vivo models and relevant tissues is required.

### 3.3. ALV-J Proliferation Evidence and Detection Methods

Multiple techniques are used to detect the proliferation of ALV-J within the host cell and provide differing points of view for confirmation of virus infection and subsequent replication of the virus [18]. All these methods are essential for virus identification, diagnosis, and research. There are three main categories of ALV-J infection detection methods. These include three groups of methods: antigen detection; nucleic acid detection; and virus isolation with titration procedures.

#### 3.3.1. Antigen Detection

Viral replication occurs inside host cells; as a result of the process of viral replication cells present viral antigens, which include gp85 and p27 [4]. The presence of even small numbers of these viral proteins can be detected with the help of IFA or an antigen capture-based ELISA [4]. An IFA uses antibodies (for example, a JE9 monoclonal Ab) to target the intracellular gp85 produced during viral infection; once attached to the viral antigen, the Ab will emit specific fluorescence when subjected to light via an epifluorescent microscope. Therefore, an IFA provides a means of identifying the presence or absence of a viral infection and identifying and assessing the type of viral infection qualitatively and quantitatively [15]. ELISA antigen capture is used to quantify or semi-quantify the amount of p27 (the viral protein forming the virus’ core) that is released into the supernatant of a cell culture or into a homogenized tissue; this antibody-based testing method has a number of advantages, including its high sensitivity and ease of implementation in the clinical setting. For this reason, antigen capture ELISAs are commonly used to diagnose and track ALV-J around the world [18].

#### 3.3.2. Detection and Amplification of Nucleic Acids

The pre-viral DNA produced by viral reverse transcription of the genomic RNA integrates into the genome of a host cell after infection of the cells by the virus. There are different ways to identify viral nucleic acids, including PCR and real-time quantitative PCR (qPCR) [18]. One way to verify that the pre-viral DNA was integrated into the host cell genome is that PCR will amplify the particular gene segments (i.e., *env* gene, *LTR* region) of the ALV-J virus from a sample of the genome taken from an infected cell [4] and qPCR will detect the presence of viral RNA or pre-viral DNA quantitatively, which can then be used to evaluate the replication of the virus over time [18]. There is also a technology called cross-primer amplification (CPA) that will allow the *gp85* gene of ALV-J to be detected very quickly and sensitively, which is suitable for point-of-care rapid testing [18].

#### 3.3.3. Virus Isolation and Titer Determination

Tissue samples (such as liver or spleen) suspected of containing infection are first ground up and sterilized via membrane filtration. The sterile tissue sample is then transferred into cell cultures (for example, CEF or DF1) that are susceptible to the virus, where they will be monitored over a certain time period for evidence of cellular damage, as well as for confirmation of the viral infection using antigen tests [4]. To determine the viral titer, the endpoint dilution assay (i.e., TCID_50_) is performed by calculating the number of wells containing positive readings from the virus-infected cell cultures at the various dilutions. The purpose of performing this assay is to assess the replication ability of a virus, and its ability to infect other cells [31].

It should be noted that the detection of viral antigens or nucleic acids does not always perfectly correlate with the presence of infectious or pathogenic ALV-J. As a retrovirus, ALV-J exhibits a high mutation rate, particularly in the *env* gene and *LTR* regions, which may reduce primer or probe binding efficiency and lead to false-negative results in nucleic acid–based assays. In addition, the continuous emergence of novel variants may allow certain strains to escape existing diagnostic systems. Therefore, comprehensive evaluation of ALV-J infection often requires the combined use of molecular, serological, and virus isolation approaches.

### 3.4. Inter-Strain Proliferation Differences and Influencing Factors of ALV-J

There are many differences between ALV-J strains in regard to their ability to proliferate inside a host cell. The way these strains differ from each other is largely determined by three factors: the genetic makeup (or genome) of the specific ALV-J strain; the type of host cell that it infects; and the culture conditions in which the ALV-Js are being cultured. The interactions of these three factors play a large role in the pathogenicity of the viral strains and their transmission efficiency. For example, viral strains isolated from chickens that are diseased (i.e., SD9901, SD9902) produce very strong positive IFA results when infecting and replicating inside CEF cells, meaning they exhibit the highest levels of viral antigen expression and fastest rates of viral replication. Conversely, viral strains isolated from healthy broilers (i.e., YZ9901, YZ9902) produce weak positive IFA results and, therefore, they exhibit lower levels of viral antigen expression and rates of viral replication These differences in the proliferation capabilities of these viruses can be attributed, in part, to the differences in the growth environment of the cells used for culturing the viruses [5,31]. In addition, vinyl strains isolated from hens that are being used for egg production (i.e., HN10PY01) have a much higher ability to replicate in primary CEF cells than do viral strains isolated from the broiler strains (i.e., NX0101). One potential explanation for this difference is due to a specific 205 nucleotide deletion in the 3′-UTR of the viral genome, which may enhance the efficiency of the viral RNA’s ability to replicate, thereby increasing the proliferation ability of the viral strain.

ALV-J proliferation, or ability to replicate, is determined by multiple factors, including the genetic characteristics of the ALV-J virus, the type of host cell that the virus infects, the culture conditions in which the virus is grown in vitro and in vivo, and the presence of any host factors that may influence the ability of the ALV-J to replicate [7]. Mutations that occur in the *env* gene of ALV-J are very important in determining the ability of the virus to proliferate [20]. In particular, mutations that occur within the receptor-binding domain of the gp85 molecule (such as N123I) serve to enhance the entry of the virus into the host cell and therefore aid in the ability of the ALV-J virus to replicate in host cells. The mutations that are found in the *LTR* region and enhancer regions have an indirect influence on the ability of the ALV-J virus to proliferate by increasing the level of transcriptional activity [31]. In terms of proliferation capability, not all host cells support the growth of ALV-J. For example, DF-1 cells are considered the best possible candidate host cell to support the proliferation of ALV-J because these cells are free of any endogenous ALV contamination and have good growth conditions. Therefore, it is important to note that the choice of laboratory host cell may affect the proliferation capacity of each batch of CEF cells used to support the growth of ALV-J. Conversely, target cells in vivo that express high levels of chNHE1 have a much higher likelihood of supporting the proliferation of the ALV-J viral strain in question than do target cells that do not express high levels of chNHE1. Generally speaking, immune cells (for example: mature dendritic cells) are very poor at supporting the growth of viruses due to their much lower levels of expression of chNHE1 or changes in the levels of expression of this receptor [10]. Specific in vitro culture conditions (such as the concentration of the CO_2_ in the environment that the viral strain is being cultured in, as well as the temperature in which the viral strain is being cultured) exert strong influences on the ability of the ALV-J virus to proliferate. Specifically, a concentration of 10% (by volume) fetal bovine serum is recommended for culturing ALV-J viral strains. Fetal bovine serum provides the cellular nutrients necessary to grow host cells and allow for the propagation of viral replication [4]. The optimal temperature for the reverse transcription, integration, and eventual assembly of progeny viral particles is 37 to 38 degrees Celsius in a 5% CO_2_ environment [7]. The maximum amounts of viral infections and viral proliferation are achieved when the target cells reach 70–80% confluence in their culture. ALV-J proliferation is mediated through several internal factors (including chicken telomerase reverse transcriptase (chTERT), doublecortin-like kinase 1 (DCLK1), and miRNAs) that modulate the proliferation of the ALV-J viral strain through pathways that mediate cellular signal transduction, the cellular cycle, and apoptosis. For example, chTERT activates the Wnt/β-catenin signaling pathway, resulting in increased growth of the host cells and decreased apoptosis and, therefore, enhanced replication of the virus [32]. When activated, DCLK1 binds to the viral SU protein and activates the MAPK signal transduction pathway, promoting the replication of the ALV-J virus [33]. MiRNAs act to regulate the expressions of the ALV-J viral genome and thereby inhibit the replication of the ALV-J virus [22].

## 4. Host Resistance Mechanisms to ALV-J and Disease Resistance Breeding

The term host resistance to ALV-J reflects the capacity of the host to defend against infection by the ALV-J virus; furthermore, the ability of the immune response of the host and/or the activation of mechanisms of the host’s genome to impede the replication of viruses and/or repair damage caused by the virus is based on the genetic composition of the host and/or the immune mechanisms of the host [11]. By understanding the host’s mechanism(s) of resistance to ALV-J, we can determine the best targets for disease-resistant breeding and control ALV-J at its source [11].

### 4.1. Natural Resistance Mechanisms of ALV-J

The host’s natural resistance mechanisms refer to the resistance traits formed during the host’s long-term evolutionary process, which do not depend on acquired immune responses. These mechanisms mainly include genetic polymorphisms, innate immune responses, and cell-autonomous defenses. They play an important role in the early stages of viral infection by inhibiting viral invasion and replication [11].

#### 4.1.1. Genetic Polymorphisms and Receptor Variations

The chNHE1 receptor on the surface of host cells is critical for ALV-J infection, and genetic variants of this receptor can directly impact a host function’s resistance to ALV-J infection via their role in ALV-J entry/replication [15]. Viral gp85 binds to the extracellular loop 1 (chECL1) region of the chNHE1 receptor. Mutations in chECL1 (e.g., W38A and E39A) interfere with viral binding to the receptor, thereby conferring resistance to ALV-J without affecting the physiological activity of chNHE1 in the maintenance of intracellular pH [15]. As chNHE1 exhibits polymorphism among different breeds of chickens, chicken breeds that are resistant to ALV-J (such as Tibetan chickens) likely possess resistant variants of chNHE1, which protect them from ALV-J infection [24]. Other genes present in the host genome may also confer resistance through regulation of viral replication or immune responses (*RFX1* and *VCAM1*), thereby forming a total genetic basis for host natural resistance [24].

#### 4.1.2. Innate Immune Response and Cell-Autonomous Defense

Upon ALV-J infection, host cells deploy a coordinated array of intrinsic defense mechanisms to limit viral replication and spread. The initial recognition of viral components by cellular sensors such as TLR7 and MDA5 triggers a signaling cascade culminating in the activation of transcription factors like IRF7 and the production of type I interferons (IFN-α/β), which establishes an antiviral state in the surrounding cells [11]. Concurrently, within the infected cell itself, critical tumor suppressor pathways are engaged. The p53 protein, for example, can induce cell cycle arrest or apoptosis to eliminate the viral replication factory [15]. Beyond classical signaling, host cells utilize post-transcriptional and epigenetic regulators for defense. Specific cellular microRNAs, including gga-miR-375 and miR-125b, have been shown to suppress ALV-J replication and associated tumorigenesis by targeting key host mRNAs involved in cell survival and proliferation [30,34]. Epigenetic regulators also play a role, as exemplified by the host protein TET2, which modulates innate immune gene expression through DNA hydroxymethylation; ALV-J counteracts this defense by actively targeting TET2 for autophagic degradation [15]. The autophagic pathway itself represents a double-edged sword, capable of degrading viral components but also potentially being co-opted by the virus. This multifaceted intrinsic response underscores the complex molecular interplay that defines the early stages of host resistance to ALV-J.

### 4.2. Artificial Creation of ALV-J Resistance

Recently, the rise of gene editing technology has allowed scientists to artificially alter chicken DNA, which allows for the development of chickens that are resistant to infection by ALV-J. Scientists can now use gene editing techniques such as CRISPR-Cas9 to make genetic changes to the viral receptor genes or the key genes involved in regulating these receptors that allow chickens to resist infection with ALV-J [17]. These advances will provide scientists with new methods to prevent and manage the spread of ALV-J [5].

#### 4.2.1. Receptor Gene Editing

Targeting the specific receptor chNHE1 of ALV-J, CRISPR-Cas9 technology can precisely edit its key functional regions (such as the W38 site of extracellular loop 1), thereby disrupting the virus’s ability to bind to the receptor and blocking viral invasion [15]. Studies have shown that after knocking out the W38 site of chNHE1, the infection rate of transgenic chicken cells to ALV-J significantly decreases, and the growth and development of the chickens are unaffected. This confirms that the method can effectively create ALV-J resistant chicken breeds [15]. Furthermore, editing other critical amino acid residues of chNHE1 (e.g., A30, V33, E39) can further optimize the resistance effect without impacting the normal physiological function of the receptor [15].

#### 4.2.2. Immune-Related Gene Editing

Genome-based strategies aimed at modulating immune-related genes have been proposed as potential approaches to enhance host resistance to ALV-J. Key immune regulators, such as IRF7, play central roles in the regulation of type I interferon production during antiviral innate immune responses. Increased activation or expression of IRF7 has been associated with enhanced interferon signaling, contributing to the control of viral replication. Similarly, pattern recognition receptors such as TLR7 and MDA5 are critical for the detection of viral RNA, leading to the activation of innate immune responses and suppression of viral RNA replication [15]. In addition, target site editing of miRNA–mRNA interactions represents an indirect genome-editing strategy to modulate host antiviral genes. For example, editing the binding site of gga-miR-23b in its target gene IRF1 prevents miRNA-mediated downregulation, thereby maintaining the antiviral activity of IRF1 and enhancing host resistance to viral infection [22].

#### 4.2.3. Editing Host Genes Critical to Viral Replication

Host organisms contain certain genes that are important for the replicating process of ALV-J (chTERT and DCLK1, respectively). Blocking the expression or function of genes through gene edits is one way to stop the replication of the virus. For example, the use of the chTERT gene edit to block the expression of chTERT, reduces the rate of proliferation and immortality of cells, and therefore reduces both the rate of replication and oncogenic results of ALV-J [2]. Editing DCLK1 prevents the viral SU protein from interacting with DCLK1 and leads to inhibition of the activation of the cell cycle, and therefore inhibits the rate of replication and release of ALV-J [33]. Thus, this technique allows for the targeting of the host factors needed for replication of the virus. Since these two methods above (targeting chTERT and targeting DCLK1), both target the necessary host factor and inhibit the replication of the virus effectively, they are considered as ideal candidates for use in stock culture generation and resistant breeding as there is also less likelihood of the virus developing resistance [15,33].

### 4.3. Current Status and Challenges of ALV-J Disease-Resistant Breeding

Recent advances in genome-wide association studies (GWAS), transcriptomic profiling, and multi-omics integration have further refined the genetic architecture underlying host resistance to ALV-J. In addition to classical resistance-associated genes such as chNHE1, RFX1, VCAM1, and chTERT, recent studies have identified novel regulatory loci and immune-related pathways involved in viral entry, replication, and host antiviral responses, highlighting the polygenic and network-based nature of ALV-J resistance [9,13,20,35]. Functional validation using CRISPR/Cas9-mediated gene editing has been extended beyond single-gene disruption, with recent reports demonstrating that targeted modification of viral receptor genes and immune regulators in DF-1 cells and transgenic chickens can significantly reduce ALV-J susceptibility without severely compromising basic cellular functions [9,14].

Importantly, contemporary studies have emphasized combinatorial and multiplex gene-editing strategies to broaden resistance spectra. Coordinated editing of multiple viral entry-related genes (e.g., chNHE1 and Tva) or key host regulatory factors has been shown to confer resistance against multiple ALV subgroups, providing a promising framework for durable antiviral breeding strategies [14,35]. In parallel, marker-assisted selection (MAS) based on high-density SNP panels and resistance-associated haplotypes remains an indispensable approach for commercial poultry breeding. Recent large-scale population studies have demonstrated that integrating genomic selection with resistance-associated markers can steadily improve flock-level resistance while maintaining production performance, thereby offering a practical and industry-acceptable alternative to transgenic approaches [13,20,36].

Despite these advances, ALV-J continues to pose significant challenges due to its high mutation rate and strong adaptive capacity. Recent molecular epidemiological analyses have confirmed ongoing genetic variation within the gp85 receptor-binding domain, enabling viral escape from host resistance conferred by receptor modification and underscoring the need for continuous surveillance of viral evolution [9,36]. Long-term monitoring of viral genomic dynamics, coupled with adaptive breeding strategies, is therefore essential to sustain the effectiveness of resistance-based control measures [35].

Furthermore, safety, ethical, and regulatory concerns remain major constraints on the large-scale application of gene-edited poultry. Recent studies have increasingly focused on evaluating the long-term physiological, reproductive, and immunological consequences of gene edits, aiming to ensure biosafety and improve public acceptance of gene-edited poultry products [14]. Collectively, emerging evidence reinforces that host resistance to ALV-J is a multifactorial and highly complex trait governed by multiple genes and interconnected pathways. Achieving durable resistance will likely require systematic dissection of host–virus interaction networks and the coordinated optimization of genomic selection, gene-editing technologies, and conventional breeding strategies to balance resistance efficacy, economic feasibility, and industrial scalability [9,13,14,20,35,36].

From a practical disease-control perspective, host resistance and disease-resistant breeding should be considered as one component of an integrated ALV-J control framework rather than a standalone solution. Effective prevention of ALV-J requires the coordinated implementation of sensitive diagnostics, continuous surveillance and purification programs, resistance-based breeding strategies, and appropriate management measures. While gene editing and genomic selection provide long-term and sustainable control potential, their application must be guided by cost–benefit analyses and risk assessment frameworks that account for breeding efficiency, biosafety, regulatory constraints, and industrial feasibility. Therefore, integrated control programs that combine molecular diagnostics, epidemiological surveillance, and resistance-oriented breeding strategies will be essential for achieving durable and economically viable control of ALV-J in modern poultry production systems.

## 5. ALV-J Research Trends and Future Directions

With the rapid development of molecular biology, immunology, and gene editing technologies, research on ALV-J has been continuously advancing. Future studies will focus on the detailed elucidation of viral pathogenic mechanisms, the development of novel prevention and control strategies, and the industrial application of disease-resistant breeding, providing more comprehensive theoretical and technical support for the effective prevention and control of ALV-J [17].

### 5.1. Detailed Study of ALV-J Viral Pathogenic Mechanisms

#### 5.1.1. Molecular Mechanisms of Virus–Host Interactions

It is necessary to conduct detailed studies on how ALV-J and its hosts interact: where within host cells do they interact? How do they act on, or affect, host cells? And what is the mechanism that regulates any downstream signaling pathway(s) resulting from this interaction? In particular, what are the mechanism(s) of action for the viral proteins (e.g., gp85, gp37, and p27) versus the host proteins (e.g., chNHE1, chTERT, and DCLK1)? A complete understanding of these interactions will provide insight into how the virus utilizes (or, “hijacks”) host cell functions at specific molecular events [30]. Systematic approaches to analyze the molecular alterations in host cells’ physiology following viral infection (using techniques such as proteomics, metabolomics, and single-cell sequencing) will provide data necessary to construct a full molecular network that characterizes each molecular interaction in this host–virus relationship and serve as a starting point for searching for antiviral drug targets [11].

#### 5.1.2. Co-Infection Mechanisms and Synergistic Pathogenesis

ALV-J is usually found in birds that have other viral (pathogen) infections together with avian leukosis virus (ALV)—J such as Marek’s Disease Virus (MDV) and/or Newcastle Disease Virus (NDV), so that the combined (synergistic) effect can help make the viruses more virulent, modify the forms of pathology produced (pathotypes), and/or potentially spread the virus more widely. Further research on the relationships between viruses during co-infection, including (for example) gene recombination and signalling regulation, the change to the host immune response, and the molecular mechanisms of synergistic pathogenesis, will help identify the epidemiological characteristics of co-infection and the damage caused by co-infection and provide the basis for the development of effective targeted prevention and control strategies against co-infection [28].

### 5.2. Development of Novel Diagnostic Technologies and Prevention Strategies for ALV-J

#### 5.2.1. Rapid and Sensitive Diagnostic Techniques

The development of rapid, sensitive, and field-deployable diagnostic technologies is essential for the effective control of ALV-J infection. Recent advances in nucleic acid amplification techniques, including cross-priming amplification (CPA) and loop-mediated isothermal amplification (LAMP), have demonstrated high sensitivity and specificity and are well suited for on-site detection without the need for sophisticated laboratory infrastructure [18,37]. In parallel, immunochromatographic assays and biosensor-based detection platforms have been increasingly explored to enable rapid screening and real-time monitoring of ALV-J infections in poultry farms [37]. Furthermore, the development of multiplex diagnostic systems capable of simultaneously detecting ALV-J alongside other clinically relevant avian pathogens is gaining attention, as co-infections are frequently observed under field conditions and may exacerbate disease severity and transmission dynamics [18,37].

#### 5.2.2. Development of Novel Vaccines

To date, no commercially available vaccine against ALV-J has been successfully developed. Traditional inactivated or attenuated ALV-J vaccines raise substantial concerns regarding biosafety, potential reversion to virulence, and insufficient protective efficacy [2,38]. Recent studies have emphasized that the high genetic variability of ALV-J, particularly within the *env* gene encoding gp85, together with the virus-induced immunosuppressive effects, represents a major obstacle to effective vaccine development [37,38]. Consequently, current research efforts are increasingly focused on next-generation vaccine strategies, including subunit vaccines based on conserved viral proteins or epitopes, virus-like particle (VLP) vaccines, and recombinant viral vector-based vaccines (e.g., adenovirus or poxvirus vectors) designed to enhance immunogenicity while improving safety profiles [2,38]. In addition, emerging evidence suggests that rational vaccine design targeting conserved T-cell epitopes and inducing robust cellular immune responses may represent a promising direction for future ALV-J vaccine development [38]. Novel platforms such as DNA and mRNA vaccines are also being proposed as potential alternatives, although their efficacy and feasibility in large-scale poultry production require further experimental validation [2,38].

#### 5.2.3. Screening and Development of Antiviral Drugs

High-throughput screening technologies have facilitated the identification of antiviral compounds targeting critical steps of the ALV-J replication cycle, including reverse transcription, proviral integration, and virion assembly and release [39]. Recent studies have further highlighted the importance of host–pathogen interaction pathways, such as chNHE1-mediated viral entry and host signaling pathways including the Wnt/β-catenin pathway, as potential antiviral targets [37,39]. In addition to conventional small-molecule antivirals, innovative therapeutic strategies based on RNA interference and CRISPR-Cas9 gene-editing technologies are being actively explored to selectively target viral genomes or essential host factors, thereby suppressing ALV-J replication at the molecular level [12,37].

### 5.3. Epidemiological Surveillance and Purification Strategies for ALV-J

#### 5.3.1. Establishment of a National Epidemiological Surveillance Network

A national epidemiological surveillance network for ALV-J should be established in the key breeding regions of the United States. Through this network, routine viral isolations and identifications, gene sequencing analyses, and serological studies will routinely be done to assist with identifying the currently circulating viral strains, their geographic distribution, host range, and mutation trends in order to provide data to assist with development of targeted prevention and control strategies [17].

#### 5.3.2. Optimization and Promotion of Purification Technology for Breeding Flocks

A significant factor in the ongoing prevalence of the virus in poultry flocks is the vertical transmission of ALV-J. To control the continued spread of the virus, it is essential to purify breeding flocks [3]. Future efforts need to be directed toward improving methods of Purifying Breeding Flocks (e.g., utilising PCR-based testing combined with the culling of all positive chickens), creating standardised protocols for Purification of Breeding Flocks, lowering costs associated with the purification process, increasing the efficiency of the purification processes and ultimately establishing a population of breeding flocks that are ALV-J negative [17].

## 6. Conclusions

ALV-J is a significant threat to the poultry industry and has made headlines recently because of its unique pathogenicity, cellular tropism, and evolution [17]. This review article presents an overview of ALV-J in terms of classification and properties, structure and genetics, pathogenicity, and epidemiology; describes the viral life cycle and replication; provides an overview of the development of natural resistance in hosts; and summarizes the progress and challenges of developing disease-resistant breeds. The progression of ALV-J pathogenicity is based on the virus–host interactions. The virus uses genetic mutations to adapt to new hosts while at the same time the host resists viral infection by utilizing genetic polymorphisms, innate immune responses, and cell-autonomous defense mechanisms. The continued investigation into these mechanisms will provide important targets for diagnosing, preventing, controlling, and developing disease-resistant breeds of chickens [4].

Recently, advances in gene-editing technology have created new opportunities to produce chickens that are resistant to ALV-J, and there remains work to be done with respect to virus mutation, safety, and commercial use. Ongoing advancements in molecular biology technology will further refine our understanding of ALV-J and will continue to produce innovative diagnostic, vaccine, and antiviral products. Through these innovations, the commercial application of developing disease-resistant breeds will continue to grow, thus providing additional support for the effective prevention and control of ALV-J and assisting in sustaining the long-term health and prosperity of the poultry industry [17].

## Data Availability

No new data were created or analyzed in this study. Data sharing is not applicable to this article.
